# Updating the CTD Story: From Tail to Epic

**DOI:** 10.4061/2011/623718

**Published:** 2011-10-15

**Authors:** Bartlomiej Bartkowiak, April L. MacKellar, Arno L. Greenleaf

**Affiliations:** Department of Biochemistry and Center for RNA Biology, Duke University Medical Center, Durham, NC 27710, USA

## Abstract

Eukaryotic RNA polymerase II (RNAPII) not only synthesizes mRNA but also coordinates transcription-related processes via its unique C-terminal repeat domain (CTD). The CTD is an RNAPII-specific protein segment consisting of repeating heptads with the consensus sequence Y_1_S_2_P_3_T_4_S_5_P_6_S_7_ that has been shown to be extensively post-transcriptionally modified in a coordinated, but complicated, manner. Recent discoveries of new modifications, kinases, and binding proteins have challenged previously established paradigms. In this paper, we examine results and implications of recent studies related to modifications of the CTD and the respective enzymes; we also survey characterizations of new CTD-binding proteins and their associated processes and new information regarding known CTD-binding proteins. Finally, we bring into focus new results that identify two additional CTD-associated processes: nucleocytoplasmic transport of mRNA and DNA damage and repair.

## 1. Introduction

Since its discovery by Fischer and Krebs in 1955 [1], the reversible phosphorylation of proteins has been implicated in the regulation of almost every aspect of cellular function, including metabolism, cell division, differentiation, signaling, and countless others. A particularly fascinating form of this regulation is employed during the transcription of DNA by RNA Polymerase II (RNAPII). Eukaryotic transcription and the concomitant pre-mRNA processing require the precise coordination between, and recruitment of, specific sets of factors at specific stages of the transcription cycle. This coupling of transcription and associated processes has been shown to be dependent on a particular feature of RNAPII, the C-terminal repeat domain or CTD [2]. Distinguishing RNAPII from its prokaryotic and eukaryotic (RNAPIII and RNAPI) counterparts, the CTD is an extension of the polymerase's largest subunit, Rpb1, and is composed of a tandem array of seven amino acid repeats with the consensus sequence Y_1_S_2_P_3_T_4_S_5_P_6_S_7_. The number of these heptad repeats varies from organism to organism and appears to correlate with genomic complexity; there are 26 repeats in yeast, 44 in *Drosophila*, and 52 in humans [3, 4]. Despite being dispensable for the catalytic activity of RNAPII, the CTD is conserved through evolution and is essential for life; for example, removing two thirds of the CTD repeats results in inviability [5, 6]. Although the function of the CTD remained elusive for several years after its discovery, research over the last three decades has confirmed its role as a selective and flexible scaffold for numerous factors involved in transcription (for reviews see [2, 7, 8]). The plastic (both in terms of conformation and susceptibility to post-translational modification) and repetitive nature of the CTD allows it to undergo a relatively well-characterized sequence of phosphorylation and dephosphorylation events during the transcription cycle (initiation, elongation, and termination), linking transcription with transcription-associated processes in a temporal manner [2, 7, 9]. The CTD has been shown to play a role in a wide variety of transcription-associated functions, and its repertoire of binding partners, modifications, and associated processes has grown rapidly over the last few years.

The CTD's unique structure, functional characteristics, and fundamental role in transcription have generated a substantial amount of interest and have made it the subject of considerable study. This research has greatly expanded our understanding of the CTD, its interacting factors, and the process of transcription in general, but as demonstrated by recent discoveries of hitherto uncharacterized CTD modifications, kinases, and binding factors/modes, there is still much that remains to be learned. As the study of gene expression and transcriptional control mechanisms expands from preinitiation into elongation, a deeper and more nuanced understanding of the CTD will likely become essential in order to deconvolute the relationships between various aspects of gene control. This is especially pertinent in terms of understanding the crosstalk between transcriptional elongation and co/post-transcriptional events, such as splicing, export, and translation. In this review, we will discuss recent developments and emerging paradigms in the study of the CTD, its modifications, binding partners, and associated processes. To further expand on an especially relevant (in terms of transcriptional control) and newly emerging CTD-associated process, we will also present an in-depth discussion of the nuclear export of RNA, with a particular focus on the interactions between the nuclear export machinery, the CTD, and transcription.

## 2. Phosphorylation of the CTD of RNAPII and the Transcription Cycle: A General Model and Its Limitations

**Figure 1:**
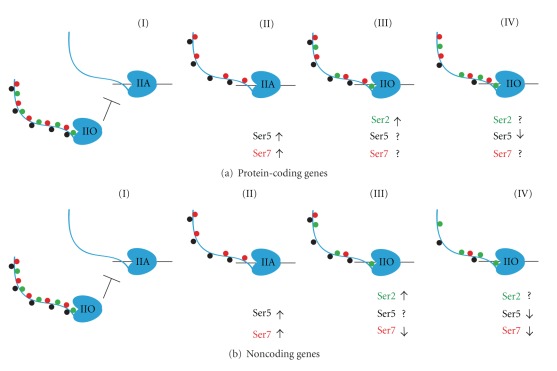
Revised “phospho-CTD cycle.” (a) (I) For protein-coding genes, RNAPII is recruited to the promoter with an unphosphorylated CTD (IIA form); moreover, it appears that CTD phosphorylation prior to preinitiation complex (PIC) formation has an inhibitory effect on transcription. (II) Upon preinitiation complex formation, the CTD is phosphorylated at the Ser5 (black) and Ser7 (red) positions. (III) During elongation, an increase in Ser2 phosphorylation (green) produces the hyperphosphorylated form of the CTD, which is probably an ensemble of singly, doubly, and triply phosphorylated heptads. (IV) As the polymerase elongates towards the 3′ end of the gene, the activity of Ser5-specific phosphatases decreases the Ser5 phosphorylation levels, while the Ser2 and Ser7 phosphate levels remain largely unchanged. (b) (I) For noncoding genes, RNAPII is also recruited to the promoter with an unphosphorylated CTD (IIA form), and CTD phosphorylation prior to preinitiation complex (PIC) formation seems to inhibit transcription. (II) Upon preinitiation complex formation, the CTD phosphorylation of Ser5 (black) and Ser7 (red) increases. (III) During elongation, Ser2 phosphorylation (green) increases, while Ser7 phosphorylation begins to decline, presumably due to the activity of a yet unidentified Ser7 phosphatase. (IV) At the 3′ end of the gene, the activity of Ser5-specific phosphatases decreases the Ser5 phosphorylation levels, Ser7 phosphorylation levels continue to decrease, and Ser2 phosphate levels remain largely unchanged.

The binding specificity of the CTD, and therefore the recruitment of particular factors, is determined by the CTD's phosphorylation state, which undergoes a series of alterations throughout the transcription cycle. Serine 2, serine 5, and more recently serine 7 of the heptad repeat have been identified as the primary targets for this transcriptionally regulated phosphorylation. In the general model of the “phosphoCTD cycle,” RNAPII is recruited for assembly at the promoter with an unphosphorylated CTD; moreover, it appears that CTD phosphorylation prior to preinitiation complex (PIC) formation has an inhibitory effect on transcription [10]. Upon PIC formation, the CTD is phosphorylated at the Ser5 and Ser7 positions; this is followed by an increase in Ser2 phosphorylation during elongation [11], yielding a CTD that contains a mix of doubly (and when considering Ser7, perhaps triply) phosphorylated repeats at Ser2 and Ser5 in the center of the gene (reviewed in [2, 9]). As the polymerase elongates towards the 3′ end of the gene, Ser5-specific phosphatases decrease the Ser5 phosphorylation levels (on noncoding genes, Ser7P levels drop as well), leaving the CTD phosphorylated at Ser2 to terminate transcription (Figure 1(a)). However, the CTD of terminating RNAPII may also be phosphorylated at Ser7 positions, as the Ser7 mark has been reported to be present at high levels throughout the entire transcription unit on many protein-coding genes [12].

Despite being highly intuitive, this gradient model of the “phosphoCTD cycle” (i.e., high-to-low Ser5P levels and low-to-high Ser2P levels as RNAPII moves across a gene, with Ser7P throughout) is oversimplified for several reasons. First, although the model accounts for variations in the general phosphorylation pattern of the CTD (recognizing the presence of gene segments with high Ser5P, high Ser5/2P, and high Ser2P levels), it fails to fully capture the highly dynamic nature of the process. The phosphorylation state of the CTD is likely to be in continuous flux throughout the entire transcription cycle, with multiple kinases and phosphatases working together to maintain specific phosphorylation patterns on particular subsets of heptads. For example, it has been recently shown that the Ser7 phosphates at the 3′ end of the gene are actually placed anew, after being removed by an unidentified phosphatase [12] (more on this below). Another complication is the existence of specific patterns of phosphorylated repeats within the CTD. Our understanding of this particular aspect of CTD phosphorylation is entangled with some of the limitations of chromatin immunoprecipitation (ChIP), the method used to characterize the phosphorylation state of the CTD at different positions within the transcription unit; thus it is convenient to discuss both of these topics together. Although ChIP can be used to measure the relative level of each phosphoCTD mark (Ser2P, Ser5P, or Ser7P) at a specific position within a gene, the repetitive nature of the CTD makes it impossible to determine exactly which of the 52 heptads (in mammals) are phosphorylated. In addition, the particular pattern of phosphorylated residues within each heptad and within sequential heptad units is also unknown; therefore, the Ser2, 5, and 7 phosphorylated CTD of elongating RNAPII is likely to be composed of heptads phosphorylated at one, both, all, and none of the relevant positions. Among the many unanswered questions concerning the detailed phosphorylation state of the CTD, an especially interesting one concerns determinism: is the phosphorylation pattern of a specific heptad at a particular position within a gene exactly the same during each transcription cycle, or does phosphorylation occur in a more stochastic manner? The answer to these questions may lie within the processivity of the CTD kinases, the extent to which they stimulate each other's activity, and the specifics of their antagonistic relationship with the CTD phosphatases.

Discussion of CTD phosphorylation patterns also raises an interesting point regarding the antibodies used to identify the post-translationally modified heptads (both in ChIP and Western blotting). It is worth reiterating that the Ser5P to Ser2P gradient model presented above is almost entirely based on the reactivity of these antibodies [13]. The most common phosphoCTD antibodies are the Ser2P-specific H5 and the Ser5P-specific H14 [14]; however, there are a multitude of other antibodies available against the CTD, including the recent and well-characterized anti-Ser2P 3E10 and anti-Ser5P 3E8 antibodies [15]. As might be expected, the reactivity of these antibodies with their specific phospho-epitopes is affected by other modifications within the heptad in question and the heptads around it. For example, the Ser2P-specific H5 antibody binds to a triheptad peptide phosphorylated on both Ser2 and Ser5 with greater affinity than to a triheptad containing only Ser2P [16]. More impressive examples are the complete lack of H14 reactivity to a single S5P in the first repeat of a diheptad peptide and the inability of 3E10 to detect an S2P followed by an S5P in the next heptad [15]. Thus, the phosphoCTD antibodies are multiheptad pattern specific, and it appears that many CTD-binding proteins are as well (see below); this correlates nicely with evolutionary studies that implicate heptad pairs as the functional unit of the CTD [17, 18]. These observations suggest that the in-depth characterization of CTD modification patterns will be important for a comprehensive understanding of factor recruitment/binding and is not a purely academic exercise. Although the ramifications discussed above (along with a few others, such as epitope masking (for a more detailed discussion see [2])) do not in any way invalidate phospho-CTD ChIP, they should be kept in mind when interpreting such data.

The general model of the phospho-CTD cycle also fails to account for exceptions to the canonical patterns of phosphorylation. The recent publication of three genome-wide studies of phosphoCTD RNAPII occupancies in yeast [12, 19, 20] has allowed for the verification of the S5P to S2P gradient model at high resolution across the entire genome. Overall, the general model appears to hold for the majority of genes [19, 20]; however, there seem to be a number of exceptions to the defined norm. The extent of these exceptions has led the authors of one of the studies to call for a reanalysis of the accepted paradigm [12], while the other two groups find that the general pattern occurs globally [20] or near globally with some stipulations [19]. It should be noted that the discrepancies between these studies might be due to the different methods used to bin/cluster genes into “average transcription units” for analysis. This binning, which is often limited by polymerase occupancy and complicated by gene length and the presence of neighboring transcription units, is especially challenging due to the compact nature of the yeast genome. One consensus that appears to emerge from the genome-wide studies is that different classes of RNAPII-transcribed genes have different CTD phosphorylation profiles. Examples include the high levels of Ser7P throughout the length of protein-coding genes (as compared to its decrease 5′ to 3′ on noncoding genes), markedly lower levels of Ser2P on noncoding genes, and enrichment/preference for specific modifications on genes for snRNAs, cryptic unstable transcripts (CUTs), stable untranslated transcripts (SUTs), genes of different lengths, and genes with different promoter classes [19, 20]. These phosphoCTD variations correlate with the different requirements for the termination and processing of distinct transcript types and make intuitive sense in the light of the CTD's role during transcription. Therefore, future versions of the “phosphoCTD cycle” will need to take such class-specific differences into account. In our revised model, we chose to separate the general cycle into two broad, but distinct gene classes: protein-coding and noncoding genes (Figures 1(a) and 1(b)). However, this revised model is still an oversimplified representation of a complex process and should be viewed as such. 

## 3. Other Post-Translational Modifications of the CTD

Lastly (in terms of complications), it has not escaped notice that modification of the CTD is not theoretically limited to the phosphorylation of Ser2, Ser5, and Ser7. Many other post-translational modifications have been observed, including phosphorylation of Tyr1 [21] and Thr4 [22] and glycosylation [23]; however, the extent and transcriptional functions of these modifications are currently unknown (for more discussion, see [8]). These “noncanonical” modifications, once fully characterized as functionally significant, have the potential to expand the CTD code further and redefine aspects of the general model; Ser7, a relative newcomer to the general paradigm, is a good example of how this can occur. 

Yet another important CTD modification, which until recently had not been directly observed to play a direct role in factor binding, is the enzymatic isomerization of the heptad repeat's peptidyl-proline bonds. Although multiple studies have suggested a role for the CTD-interacting peptidyl-prolyl *cis/trans* isomerases (Ess1 in yeast and Pin1 in humans) in transcription and CTD phosphorylation [24–26], all of the structures of CTD-substrates/CTD-binding protein complexes revealed the CTD proline residues to be exclusively in the more energetically stable, and therefore predominant, *trans* state. This changed last year when two structural studies found that the Ser5-specific CTD phosphatase Ssu72 bound to the *cis* conformation of an Ser5-Pro6 motif within the heptad repeat [27, 28]. Concordantly, the activity of the proline isomerase Ess1 was found to facilitate the rapid dephosphorylation of the CTD by Ssu72 *in vitro*, suggesting that this *cis/trans* interconversion plays a role in the fine-tuning of the phosphorylation state of the CTD [27]. These findings have broad implications for CTD biology, both by increasing the number of distinct CTD states and serving as a regulatory mechanism for CTD phosphorylation. However, it still remains to be determined whether proline isomerization is a general property of RNAPII transcription or if it is gene specific [27], a distinction that may apply to other types of modifications as well. 

A good example of a transcript class-specific CTD modification is the newly discovered methylation of an arginine (R1810) in heptad 31 of the human CTD [29]. As an apology for the arginine (one of two in the human CTD), it should be noted that while the first 26 repeats of the human CTD conform strongly to the consensus sequence (YSPTSPS), there is significant divergence from the consensus in the C-terminal half of the CTD [30]. It has been previously postulated that the various noncanonical heptads (and even particular segments of the CTD; such as the N- and C-termini [31]) may have specific functions, and this arginine methylation seems to be a case in point (for further discussion, please see [30]). Mediated by the methyltransferase CARM1 and inhibited by Ser5 and Ser2 phosphorylation, the methylation appears to repress the expression of snRNAs and snoRNAs in a general manner [29]. This and other modifications of the noncanonical heptads may serve as a discriminatory mark for RNAPII recruited to particular genes or transcript classes. It should also be noted that Ser7P is currently thought to be transcript class-specific CTD modification, as Ser7 to alanine mutations in the CTD cause a defect in snRNA transcription while having little effect on protein-coding genes [32]. However, the ubiquitous nature of Ser7P on protein-coding genes, along with the finding that Ser7 is enriched on RNAPII within introns [19], argues for some (perhaps more subtle) functional role for Ser7P on most transcription units.

Thus, the general “phosphoCTD cycle” has given way to a “CTD code” of staggering complexity, one that we are just beginning to explore in detail. This complexity reflects the vast number of different genes, processing events, and transcriptional programs that RNAPII must coordinate. Although the segmented gradient model has proven to be very useful for conveying the CTD's principal function during RNAPII transcription, as our understanding of the CTD and associated processes improves, it is likely to undergo drastic changes in the near future. Understanding the nuances of this CTD code will be imperative to understanding the link between transcription and cotranscriptional events and to perhaps eventually unlock the therapeutic potential of the CTD.

## 4. The CTD Kinases

The specific phosphorylation events within the heptad repeat are mediated through the activity of a transcription-associated subset of cyclin-dependent kinases (CDKs) known as the CTD kinases. Unlike their cell cycle counterparts, the CTD kinases form complexes with members of the noncycling “transcription cyclin” family and are active throughout the cell cycle. Nevertheless, CTD kinase activity is tightly regulated through a variety of mechanisms, including selective recruitment, binding by kinase-associated factors, and sequestration by inhibitory factors. Although somewhat promiscuous *in vitro* (for example, the Ser2-specific CTD kinase Ctk1 can phosphorylate both Ser2 and Ser5 *in vitro* [16]), *in vivo* the CTD kinases are selective for particular heptad residues (Ser2, 5, and 7) and stages of transcription. Thus, the various CTD kinases are most conveniently presented in the context of a segmented transcription cycle; however, it should be made clear that in reality the various kinase activities lack the clearly delineated boundaries that such a presentation suggests.

Although we will not be discussing them in detail, it should be kept in mind that the CTD kinases function in conjunction with the more enigmatic CTD phosphatases (several of which have been characterized in yeast, including Fcp1 [33], Ssu72 [34, 35], and Rtr1 [36]; see [7] for a review and [9] for further discussion). 

### 4.1. Initiation and the Promoter: CDK7 and CDK8

The phosphorylation of CTD Ser5 and Ser7 residues during the formation of the preinitiation complex is mediated by the CTD kinase subunit of the general transcription factor TFIIH: Kin28/Ccl1 in yeast and CDK7/CyclinH in metazoa [11, 13, 37–39]. In an elegant interplay, the kinase activity of Kin28 is stimulated by the mediator coactivator complex, which binds to, and delivers, unphosphorylated RNAPII to the promoter [40]. The resulting phosphorylation of the CTD leads to the dissociation of mediator [41]; thus after fulfilling its function, mediator is able to use the CTD and Kin28 to induce its own release from transcriptionally active RNAPII. Intriguingly, a subpopulation of the mediator complex has been found to include an extra module that contains the CDK8/CyclinC (Srb10/Srb11 in yeast) kinase/cyclin pair. Initially discovered as a suppressor of a CTD truncation [42], CDK8 has emerged as the only CTD kinase to be implicated in the repression of transcription. Concordant with the process of mediator release, one of the mechanisms by which CDK8 has been proposed to execute its inhibitory activity is through the premature phosphorylation of the CTD (prior to PIC formation) and inactivation of the CDK7/CyclinH complex [10, 43]. Although historically the focus has been on CDK8's role as negative regulator of transcription, increasing numbers of studies are finding that CDK8 can also play a positive role in transcriptional activation. Therefore, a complete understanding of the function of CDK8 in CTD phosphorylation and transcription remains elusive and is likely to be a topic of much research in the near future (for more details and a comprehensive review, see [7, 44]).

Once thought to be essential for promoter clearance, the activity of Kin28 has been shown to be dispensable for global gene transcription [45, 46]. Despite this, Kin28 has been found to enhance polymerase progression through long genes in yeast (over 2 kb), suggesting that it plays a role in transcriptional elongation or in the inhibition of premature termination [19]. CDK7 also takes part in the phosphorylation and activation of other CDKs (see [47] for a review); however, its (and Ser5Ps) most clearly defined transcriptional role is the recruitment of the 5′ end capping machinery. Not only does this ensure the proper processing of the nascent mRNA, it has also been shown to mediate the recruitment of the Ser2 CTD kinases in some organisms (either directly or through recruitment of the capping machinery) [48–50]; this suggests that phosphorylation of Ser5 plays a role in triggering the onset of Ser2 phosphorylation.

### 4.2. The Elongation Phase: Ctk1, Bur1, and Their Metazoan Counterparts

Subsequent to Kin28 activity at the promoter, phosphorylation of Ser2 of the CTD heptad occurs downstream of the transcription start site (TSS) and coincides with RNAPII entry into productive elongation. Coupled with the activity of Ser5P-specific phosphatases (Rtr1 in yeast [36]), this leads to a transition from high Ser5P to high Ser2P (as characterized by ChIP). The Ser5P to Ser2P crossover point, defined as the point at which the ChIP signals for the two CTD marks cross, is on average ~450 bp downstream of the TSS and appears to be independent of the overall gene length [19]. This implies that the dynamics of Ser2 and Ser5 phosphorylation are not scaled to gene length; however, the significance of the crossover point in terms of the actual phosphorylation state of the CTD is obscure.

In *Saccharomyces cerevisiae (Sc)*, the phosphorylation of Ser2 is primarily mediated by CTDK-I, a three subunit enzyme (consisting of Ctk1, a CDK homologue; Ctk2, a cyclin homologue; and Ctk3, whose function is unknown) [51, 52]. Although it is responsible for the bulk of Ser2 phosphorylation *in vivo, *Ctk1 is not essential for viability or for transcriptional elongation. The CTD kinase activity of Ctk1 has been linked to several transcription-associated processes, including the recruitment of the Set2 histone methyltransferase [53, 54], 3′ end processing [19, 55, 56], and termination (reviewed in [9]). In addition to its role as a CTD kinase, Ctk1 (independent of its kinase activity) has also been shown to be involved in the dissociation of basal transcription factors from RNAPII [57]. Despite having a principal role in CTD Ser2 phosphorylation, Ctk1 is not the only Ser2 kinase in yeast; it coexists with the essential Bur1 kinase (which consists of the CDK homologue Bur1 and the cyclin Bur2) [58]. While it has been proposed that Bur1's primary transcription-related substrate is the elongation factor Spt4/5 [59, 60], rather than the CTD, recent evidence indicates that Bur1 binds to the Ser5P CTD and contributes to Ser2 phosphorylation during early elongation, possibly stimulating subsequent Ctk1 activity [49, 61]. As mentioned previously, Bur1 has also been found to exhibit an elongation phase Ser7 kinase activity, which appears to counteract the activity of a yet unidentified Ser7 phosphatase [12]. Another pair of Ser2 elongation kinases is also present in the fission yeast *Saccharomyces pombe (Sp)*, where Lsk1, the *Sp *orthologue of Ctk1, has been shown to be responsible for the bulk of Ser2 phosphorylation, while *Sp* CDK9, the *Sp *orthologue of Bur1, is able to phosphorylate both the CTD and Spt5 [50, 62].

Until recently, higher eukaryotes appeared to have only one Ser2 CTD kinase: P-TEFb (which is composed of CDK9 and cyclinT). P-TEFb is able to phosphorylate both the Ser2 position of the CTD and the elongation factor Spt5 and is essential for transcriptional elongation [63–65] (for detailed discussions, see [66–68]). The substrate specificity of P-TEFb, coupled with its equal sequence similarity to both Bur1 and Ctk1, has led to the proposal that P-TEFb reconstitutes the activities of both yeast kinases in higher eukaryotes [69]. However, there was some evidence that this may not be the case; two evolutionary studies concluded that while *Sc* Bur1 is the closest *Sc* relative of metazoan CDK9 proteins, *Sc* Ctk1 is actually more closely related to another set of relatively little-studied metazoan CDK proteins [70, 71]. Based on these evolutionary studies, work in our lab has characterized the previously unstudied *Drosophila* CDK12 (dCDK12) and little-studied human CDK12 (hCDK12) as elongation phase CTD kinases and the metazoan orthologues of yeast Ctk1 [72]. Unlike most other cell cycle and transcriptional CDKs, CDK12 (and CDK13, a highly related paralogue absent in Drosophila but present in many “higher” organisms) contains splicing factor-related structural features (RS domains) and has been previously implicated in the regulation of alternative pre-mRNA splicing [73–77]. Although both CDK12 and CDK13 manifest CTD kinase activity in *in vitro *kinase assays, only CDK12 seems to have an effect on global CTD phosphorylation *in vivo*; thus CDK13's role in transcription (assuming one exists) remains elusive [72]. In terms of the cyclin partner of CDK12, our lab found that endogenous dCDK12 associates with cyclinK, a Ctk2-like cyclin that has been previously characterized as an alternative partner for CDK9 [78]. These findings are inconsistent with previous reports that CDK12 and CDK13 interact with the L class cyclins [74, 75]; thus, whether cyclinK is the cyclin partner of human CDK12 and CDK13 remains to be determined. As of this paper, other than our initial characterization, there have been no published studies of CDK12 and CDK13 in the context of transcription and transcriptional elongation, thus much remains to be learned about these kinases. Intriguingly, the depletion of dCDK12 affects the phosphorylation state of the CTD without affecting RNAPII occupancy (BB and ALG, unpublished); therefore, CDK12 might prove to be a useful tool for studying the links between CTD phosphorylation patterns and transcription elongation-associated processes in higher eukaryotes.

A final point regarding the CTD kinases relates to their therapeutic potential. As the CTD kinases are involved in the coupling of various signaling pathways to transcription and RNA processing events, they play important roles in the regulation of cell growth, proliferation, and survival. Thus, targeting these kinase activities may be potentially useful for the treatment of human diseases and cancer [79]. In fact, the CDK inhibitor flavopiridol, which targets P-TEFb, is used for the treatment of some forms of leukemia, and P-TEFb has been implicated in HIV replication [80, 81]. The emerging links between the CTD and DNA repair/genomic stability (see below), combined with the fact that ~15% of disease-causing mutations are a consequence of the misregulation of alternative splicing [82] (a function associated with both the CTD and CDK12/13, see below), suggest that a more comprehensive understanding of the CTD and its kinases could have broad medical implications in the future.

## 5. CTD Functions 

The CTD has been implicated in a broad spectrum of transcription-associated functions, and its collection of binding partners has continued to expand over the last few years. Important target processes include mRNA (and snRNA) capping, splicing, 3′ end processing, termination, and more recently nuclear export (discussed below). In terms of non-RNA processing-associated events, the CTD has been shown to play roles in transcriptional activation, cotranscriptional chromatin modification, chromatin remodeling, and genome stability. Of course, the analysis of the CTD's role in each specific function is very challenging, as many of the CTD-mediated transcriptional processes are interlinked. For example, capping has been shown to influence both the splicing of the first intron and 3′ end processing [83–87]; splicing of the last intron affects 3′ end processing and vice versa [88–90]; and alternative splice site choices are affected by the cotranscriptional histone modifications at splice site junctions [91–93]. While these interactions demonstrate the high degree of coordination involved in mRNA synthesis, they unfortunately complicate the interpretation of functional studies. In a broad sense, it is fair to state that exactly how S2, S5, and S7 phosphorylation affect initiation, elongation, and termination remains poorly understood. Despite the lack of a universal understanding, particular aspects of CTD function have been characterized to an impressive level of detail, and many of the more enigmatic functions are becoming better understood through continued investigation. In the next few sections, we present a rather cursory overview of the functions of the CTD (for other reviews, see [2, 7, 8]) before moving on to a discussion of two newly emergent CTD-related functions: mRNA export and DNA repair/genomic stability.

### 5.1. 5′ Capping

As mentioned previously, one of the most clearly recognized functions of the CTD is its involvement in the 5′ end capping of mRNA through the recruitment of the capping machinery. The modification of the 5′ end of the RNA with the 5′ 7-methyl guanosine cap is unique to RNAPII transcripts and occurs just after the transcript clears the polymerases exit channel [94, 95]. Transcripts made by a CTD-less RNAPII were found to be inefficiently capped, leading to the characterization of the physical interaction between the capping enzymes and the phospho-CTD [96–98]. Subsequent studies showed that the capping enzyme associates with the 5′ end of genes *in vivo*, which correlates with the enzyme's function, and that this association is dependent on phosphorylation of Ser5 of the CTD [11, 37, 46]. In addition to raising the local concentration of the capping enzyme near the exit channel, the CTD has also been shown to stimulate its activity. An example of a phosphorylation state-specific function, mammalian guanylyltransferase was found to bind to both Ser2P and Ser5P synthetic heptad repeats but was allosterically activated only by Ser5P [99]. The interaction between the 5′ capping machinery and the CTD has also been investigated structurally, resulting in some interesting insights. The crystal structure of the *Candida albicans* guanylyltransferase Cgt1 complexed with a synthetic Ser5P four heptad repeat peptide revealed that the CTD binds within an extended docking site on the enzymes surface using two nonadjacent heptads, and a full-heptad repeat was looped out away from the interaction site [100]. This looping not only demonstrates the inherent flexibility of the CTD but also suggests that by binding two remote heptads, CTD binding factors may be able to loop out large portions of the CTD. This looping could potentially result in the formation of novel structural motifs, which could in turn serve as binding sites for other CTD binding factors, leading to organized, sequential binding [7]. Whether this actually occurs is still an open question; however, recent studies have shown that the binding of some well-known 3′ end processing factors to the CTD appears to be cooperative in nature [101].

### 5.2. 3′ End Processing

Another well-recognized function of the CTD is its role in 3′ end processing and termination (for reviews, see [102, 103]). Analogous to capping, transcription by a CTD-less RNAPII was shown to affect both processes, and cleavage and polyadenylation factors were found to bind to the phospho-CTD [104–110]. Accordingly, inhibition of Ctk1 in yeast has been shown to decrease the efficiency of cleavage at poly(A) sites [56] and result in the disruption of polyadenylation factor recruitment to the 3′ end of the gene [55]. A genome-wide analysis has also shown that depletion of Ctk1 using a tetracycline-repressible degron mutant causes a “pileup” of polymerases at the poly(A) site in a subset of genes with good consensus poly(A) sequences [19]. This increase in RNAPII occupancy suggests that improper CTD phosphorylation at these sites can result in a strong transcriptional pause that is perhaps due to the rate-limiting recruitment of a specific factor. Strongly linked to polyadenylation, termination has also been reported to be affected by the CTD [108]; in addition, Rtt103, a component of the termination complex, has been shown to bind Ser2P CTD [111]. However, the role of CTD modification in termination is not yet well understood (see [9, 103, 112] for further discussion). Intriguingly, it has been observed that the recruitment of one well-recognized CTD binding 3′ end processing factor, Pcf11 (which preferentially binds to Ser2 phosphorylated CTD), does not directly correlate with the level of Ser2 CTD phosphorylation [20, 55, 106]; analysis by ChIP indicates that Pcf11 is recruited mainly at the poly(A) site, while Ser2P levels rise throughout the coding region. Potential explanations for this phenomenon highlight some of the interesting complications surrounding phospho-CTD factor recruitment. Perhaps Pcf11 requires a certain threshold of Ser2P or both Ser2P and an external signal, such as the presence of the newly synthesized polyadenylation site, the unmasking of particular CTD epitopes, or the presence additional factors (Pcf11 CTD binding was recently shown to be cooperative [101]). Other considerations include the remodeling of the CTD via a pattern-specific change (such as the formation of Ser2P only heptads) or modifications that are undetectable by ChIP. With regards to the latter and the previous discussion on proline isomerization, Pcf11 was reported to specifically recognize three neighboring *trans* prolines within a mixed population of *cis-trans* isomers [113]. It is likely that the recruitment of many other CTD-binding factors is also mediated through multiple mechanisms and dependent on the satisfaction of particular sets of conditions.

### 5.3. snRNA Processing

One of the relatively more recently discovered functions of the CTD is its role in the transcription and 3′ end processing of snRNAs [114, 115]. Even though they are transcribed by RNAPII, snRNAs are unlike most coding transcripts; they do not undergo splicing or polyadenylation and instead rely on a conserved 3′ box RNA processing element downstream of the coding region for proper 3′ end processing and termination [116]. The 3′ end processing of snRNAs has received a lot of recent attention, as it is currently the only specific function attributed to the phosphorylation of Ser7 of the heptad repeat [32]. Seemingly dispensable for viability and expression of protein-coding genes, Ser7 has been found to be essential for endogenous snRNA gene expression. This requirement for Ser7 phosphorylation was subsequently linked to the integrator complex [32], a large CTD-associated multiprotein complex involved in snRNA 3′ end processing [117]. Further characterization of the CTD-integrator interaction not only revealed that both Ser2P and Ser7P were required but also that efficient binding required a specific arrangement of the modifications [118]. Screening of synthetic diheptad repeats revealed that although maximal binding was achieved with both Ser2 and Ser7 phosphorylated repeats, the minimal interaction domain consisted of a Ser7P on the first heptad followed by a Ser2P on the second heptad; any other combination of two phosphates was insufficient for integrator binding [118]. These findings lend further support for the pattern-specific binding of CTD-associated factors and, coupled with the fact that integrator is specific to snRNA genes, also suggest that the appropriate Ser2P/Ser7P patterns may be snRNA gene specific (although many other discriminatory mechanisms could be at play, such as the previously discussed methylation of R1810).

### 5.4. Histone Modifications

Tying together two important aspects of gene expression and transcriptional coordination, the CTD has also been shown to be involved in the cotranscriptional modification of histones and remodeling of chromatin structure. Although a full discussion of histone modifications and the histone code hypothesis is beyond the scope of this paper, the significance of these processes cannot be understated: they play integral functions in almost every aspect of gene expression and regulation (for a review, see [119]). Here, we will only give a very brief overview of some CTD-related functions and a short list of new developments.

Providing the first clear link between the CTD and histone modification, the yeast Set1 and Set2 methyltransferases were found to be recruited to actively transcribed genes at specific stages of the transcription cycle (5′ end versus interior of the gene, resp.) through interactions with the Ser5P (via the PAF complex for Set1) and Ser2/5P CTD [120–123] (see [124] for a review). Thus, Set1's activity, the methylation of histone H3 at the K4 position, peaks near the promoter, while Set2's methylation of H3 at K36 occurs downstream in the coding region. The H3K36 trimethylation mark can be used to identify transcriptionally active genes and has been shown to suppress inappropriate transcription from cryptic promoters, which was initially thought to occur through the recruitment of the histone deacetylase Rpd3S [125, 126]. Surprisingly, recent studies have found that Rpd3S is actually recruited via direct binding to the phospho-CTD; although H3K36 trimethylation is dispensable for Rpd3S recruitment, it appears to be required for activation of its deacetylation activity [127, 128]. Intriguingly, Set2 is capable of H3K36 dimethylation independent of its CTD-interacting SRI domain or Ctk1 [54], implying that only one specific aspect (H3K36 trimethylation) of Set2's activity is regulated by interaction with the phospho-CTD. Another interesting chromatin-related CTD binding factor is the histone H3 chaperone and transcription elongation factor Spt6 [129]. Spt6 has been shown to bind the Ser2P CTD through a tandem SH2 domain [130] and interact with the multifunctional elongation factor IWS1/Spn1, an Spt6-interacting factor that associates with the nuclear RNA export factor REF1/Aly (and Yra1 in yeast (ALM and ALG, unpublished)) and possibly facilitates nuclear export [129]. In addition to its association with REF1/Aly, IWS1/Spn1 has been recently found to be required for the optimal loading of the mammalian Set2 (HYPB/Setd2) in the coding regions of several genes [131], linking nucleosome reassembly with elongation-coupled H3K36 trimethylation *in vivo*. Several other recently characterized chromatin-related CTD interactions include the recruitment of the chromatin-remodeling factor CHD8 [132] and the FACT histone chaperone via HP1 [133]. The number of recognized chromatin-associated CTD interacting factors is likely to grow rapidly over the next few years as our understanding of both the processes of, and the relationship between, CTD phosphorylation and histone modification improves.

### 5.5. Splicing

One of the more intriguing but relatively poorly characterized functions of the CTD is its involvement in cotranscriptional splicing (for reviews, see [134, 135]). Although neither active transcription nor the CTD is absolutely required for splicing (presynthesized mRNAs can be spliced by injection into *Xenopus* oocytes or by incubation with nuclear extracts [136]), experimental evidence accumulated over the last three decades implicates the CTD as a key player in the coupling between the two processes. The link between the CTD and splicing was first proposed in the early 90s [137], and in the mid-to-late 90s, the hyperphosphorylated RNAPII was shown to associate with the SR (Serine/Arginine rich) family of splicing factors and with components of the splicing machinery [138–140]. Concordantly transcription by a CTD-less RNAPII was shown to result in low splicing efficiency *in vivo* [108], and the addition of an anti-CTD antibody or exogenous expression of CTD peptides resulted in the accumulation of unspliced transcripts [140] and the nuclear reorganization of splicing factors [141]. In what could be considered the reciprocal experiment, it was also shown that isolated CTD fragments and purified phosphorylated RNAPII were able to activate splicing reactions *in vitro* [142, 143]. In addition to the SR-like CTD-associated factors (SCAFs) [140, 144], several other CTD-binding splicing factors have been identified, including the yeast U1 snRNP component Prp40 [145] and the mammalian splicing factors CA150 (TCERG1) [146], PSF, and p54/NRB [147]. A recent addition has been the splicing factor U2AF, which in an *in vitro* complementation assay was shown to be recruited to the CTD in complex with another splicesome component, Prp19, in order to overcome a weak polypyrimidine-binding tract in an IgMA3 substrate [148]. Although alternative splicing has been directly demonstrated to be affected by the presence of the CTD [149], the fact that RNAPII elongation rate has also been implicated in splice site choice [150, 151] has made it difficult to determine how much of the effect is due to the specific recruitment of splicing factors to the CTD and how much is due to changes in the elongation rate (kinetic coupling) or other processes. One study that merits specific mention is that of Batsche et al., who reported that the inclusion of a set of alternative exons in the middle of the CD44 gene was dependent on the site-specific accumulation of RNAPII eoccupancy induced by the catalytic subunit of the SWI/SNF chromatin-remodeling complex [152]. Surprisingly, this “stalled” RNAPII exhibited a SWI/SNF-dependent switch of the RNAPII phosphorylation status from the elongation characteristic Ser2P to the more promoter characteristic Ser5P, which perhaps creates a barrier to further elongation (for a detailed discussion regarding kinetic coupling please, see [134]). In addition to its general role in the recruitment of splicing factors, it has also been suggested that the CTD may function as a molecular tether for distant splice sites within the mRNA. This would be accomplished through the binding of the nascent 3′ splice site to a CTD-associated splicing factor, thus effectively immobilizing it near the polymerase mRNA exit channel in anticipation of the cognate 5′ splice site. Enhancement of the local concentration of the splice sites would then dramatically increase the efficacy of the splicing reaction (see [135]). This tethering model is especially attractive in higher eukaryotes in which the intron lengths of many genes exceed thousands of base pairs. Although studies have demonstrated that mRNA tethering is likely [153], it has yet to be directly linked to the CTD and its phosphorylation.

Although it is not the only CDK kinase to have been implicated in the regulation of both transcription and alternative splicing (CDK11/cyclinL has been shown to affect both processes but lacks a reported CTD kinase activity [154, 155]), CDK12's CTD kinase activity coupled with its structural and functional characteristics places it directly at the juncture between transcription, the CTD, and splicing. The N-terminal domains of both CDK12 and its more enigmatic paralogue CDK13 contain arginine/serine (RS) dipeptide-rich segments, which are characteristic of splicing factors and splicing factor regulators and are believed to be important for protein-protein interactions [156, 157]. Much like other RS domain-containing proteins and most factors involved in pre-mRNA splicing, CDK12 and CDK13 were found to exhibit a punctate pattern of localization in the nucleus, superimposed on a more even distribution throughout the nucleoplasm. The punctate localization points appear to represent “nuclear speckles” [76, 77], commonly thought to be sites of splicing factor storage [158]. In accordance with these structural features and localization, *in vivo* splicing assays using reporter genes have demonstrated that the ectopic overexpression or depletion of CDK12 and CDK13 modulates alternative splice site selection [74, 75, 77]. In addition, CDK13 was observed to affect HIV splicing in a Tat-dependent manner [73]. These splicing effects were postulated to be a consequence of CDK13-mediated phosphorylation of the canonical SR splicing factor ASF/SF2 [76], but all of the extant studies utilized ectopic overexpression of CDK12 and CDK13, usually without their potential cyclin partners, in assay systems that can detect splice site changes but are unable to provide mechanistic insights. Moreover, many of the results appear to argue against the direct phosphorylation of specific splicing factors by CDK12 and 13; for example, the N-termini of CDK12 and 13 are able to affect splicing independently of the kinase domains; and one study has reported that the phosphorylation of ASF/SF2 by CDK13 appears to be indirect [74]. Complicating analyses of this type is the fact that the overexpression of virtually any SR protein will have effects on splicing via sequestration of other SR proteins (and SR protein-binding partners), competition for SR phosphorylating and dephosphorylating factors, and the occurrence of other nonspecific and unforeseen events; thus, it is difficult to amalgamate the current data into an overall consensus. Despite these caveats, the unusual structures of CDK12 and CDK13, coupled with their ability to modulate splice site choices in *in vitro* assays, make it tempting to speculate that the two kinases may serve as a central link between the processes of transcription, CTD phosphorylation, and splicing; however, whether such a link exists is still undetermined.

### 5.6. The CTD and mRNA Export

While the connection between mRNA processing and the CTD has been established, recent studies have begun to investigate CTD involvement with the last step in mRNA production: formation of an export competent messenger ribonucleoprotein particle (mRNP). While this area of research has yet to mature, it is known that mRNAs are exported through a Ran-GTP-independent pathway that involves a specific set of conserved export receptor and adaptor proteins. There appears to be one universal receptor for mRNA export, Mex67/Mtr2 in yeast and TAP/p15 in mammals, which interacts with the mature mRNP and the nuclear pore complex (NPC) to facilitate export. The export receptor functions in conjunction with the export adaptor proteins, which cotranscriptionally associate with the nascent mRNA. There are two main mRNA export adaptor proteins, Yra1/ALY in the transcription and mRNA export (TREX) complex and Sac3 in the TREX-2 complex. While export mediated by TREX-2 and Sac3 has been shown to be coupled to chromatin modification through Sus1, a common factor in both TREX-2 and the Spt-Ada-Gcn5 acetyltransferase (SAGA) complex (reviewed in [159, 160]), we will focus on the novel connection between mRNA export by Yra1 and the CTD. 

TREX was the first characterized transcription export complex and consists of the THO complex of elongation and hyperrecombination-related factors, including Hpr1, Tho2, Thp1, and Mft1; the export adaptor protein Yra1/ALY; the RNA helicase Sub2/UAP56; a protein of unknown function, Tex1 [161]. Yra1/ALY has been proposed to link aspects of mRNA splicing and processing to mRNA export based on its interactions with Sub2/UAP56 [162, 163] and Pcf11 [164] and the observation that ALY may be associated with the exon junction complex in mammals [165–167]. The Hpr1 subunit of THO has been hypothesized to play an important role in Yra1 and Sub2 recruitment because *Δhpr1* yeast displays a decrease in the levels of both Sub2 and Yra1 occupancy on certain genes [168]; however, as deletion of *HPR1* has been shown to affect transcription elongation, this decrease in Yra1 and Sub2 occupancy may be due to an elongation defect rather than a direct effect of Hpr1 on the export proteins [169].

In a previous study from our lab, a proteomics screen in yeast identified Yra1 as a putative phospho-CTD-associated protein (PCAP) [170]. We have since characterized the phospho-CTD- (PCTD-) binding activity of this export adaptor and demonstrated (*via* ChIP) that partial deletion of its phospho-CTD interaction domain (PCID) leads to a near loss of Yra1 association with active genes (MacKellar and Greenleaf, In press (*J. Biol. Chem.*)). We therefore think that either Yra1 is responsible for recruiting the rest of TREX to active genes via the CTD or Yra1 is recruited to active genes independently of THO. Further chromatin immunoprecipitation studies using additional Yra1 variants that do not bind the CTD are needed to further clarify the role of the CTD in Yra1 function and the dependence of the rest of TREX on Yra1.

We also found that the PCID of Yra1 contains an RNA recognition motif (RRM). While RRMs are known to be versatile-binding domains that mediate both protein-nucleic acid and protein-protein interactions (see [171, 172] and references therein), until this year no protein had been found to use an RRM for PCTD binding. However, a recent study on spliceosomal factor U2AF65 indicated that its noncanonical RRM (also called a U2A homology motif (UHM)) might also mediate an interaction between the splicing factor and the PCTD [148]. We propose that there is a class of factors that use its RRM as a dual-purpose domain first for associating with the transcription elongation complex *via* PCTD binding and then for associating with the nascent transcript through RNA binding. Structural studies on Yra1 in complex with the PCTD are necessary to examine the role and binding mode of the RRM in this interaction.

**Figure 2:**
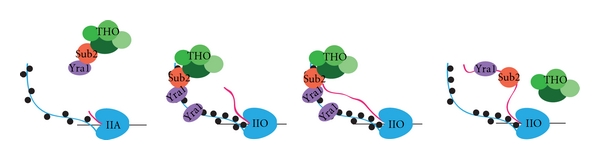
CTD-mediated recruitment of Yra1. Yra1 (possibly in complex with THO and Sub2) does not bind to Ser5P containing CTD and is not observed at high level at the 5′ end of genes. Yra1 is recruited to active genes during elongation when the CTD is doubly phosphorylated on Ser2 and Ser5. As it is not known whether Yra1 can bind both the CTD and TREX, it is modeled in both contexts. Yra1 remains bound to the CTD until the nascent mRNA reaches a certain length or reaches the CTD-bound Yra1; this would avert premature export attempts. At the 3′ end of the gene, Yra1 dissociates from the CTD, either because the phosphorylation pattern changes to predominant Ser2 phosphorylation (in which Yra1 does not bind) or because Yra1 binds the nascent mRNA with higher affinity.

Based on our discovery that the CTD is involved in recruiting Yra1 to genes, we have revised the model of Yra1 cotranscriptional recruitment to include the CTD (Figure 2). We propose that Yra1 (possibly in complex with THO and Sub2) is recruited to active genes during elongation when the CTD is doubly phosphorylated on Ser2 and Ser5. Because we do not yet know whether Yra1 can bind both the CTD and TREX, it is modeled in both contexts. As the nascent mRNA grows, Yra1 remains bound to the CTD, and this may prevent Yra1 from blocking the activities of other mRNA processing factors, averting premature export. At the 3′ end of the gene, Yra1 dissociates from the CTD, because either the phosphorylation pattern changes to predominantly Ser2 phosphorylation (which Yra1 does not bind) or Yra1 binds the nascent mRNA with higher affinity. This model is still largely speculative, and further experimentation is required to test the hypotheses it represents.

### 5.7. The Next Frontier in CTD Research: DNA Damage and Repair

Results connecting transcription/RNA processing with recombination and DNA damage repair have been obtained in bacteria, yeast, and mammals (reviewed in [173]). For example, defects in mRNA splicing (by ASF/SF2 in mammals) and packaging (THO/TREX and TREX-2 in yeast) have been linked to genomic instability and hyperrecombination via R-loop formation [174–176]. A potential involvement of the CTD in repair/recombination is suggested by the observations that mutations in the subunits of the CTDK-I kinase render yeast sensitive to DNA-damaging agents and that DNA damage leads to alterations in the phosphorylation pattern of the CTD [177]. Moreover, recent work hints that the CTD and its associated proteins play a role in sensing DNA damage and promoting repair. 

For example, Bennett and colleagues used the diploid yeast deletion strain collection to identify a large number of genes whose homozygous deletion leads to ionizing radiation (IR) sensitivity [178, 179]. We have found that a significant number of these genes encode phospho-CTD-associating proteins (PCAPs), thus linking IR damage repair to the phospho-CTD (Winsor et al., *in prep*). A different kind of damage repair, as signaled by mitotic recombination, also appears PCTD linked since diploid yeast deleted for *CTK1* (*ctk1Δ/ctk1Δ* strains) displays reduced rates of spontaneous mitotic recombination at several loci (Winsor et al., *in prep*). Thus, the PCTD appears to be involved in processes that maintain genome stability. 

In a related vein, it was shown recently that mammalian RecQ5 protein, a putative “antirecombinase,” is associated with RNAPII on active genes [180, 181]. *In vitro* experiments showed that RecQ5 binds directly to the elongation-associated phospho-CTD *via* a Set2 Rpb1-interaction (SRI) domain; moreover, deletion of the SRI domain resulted in loss of RecQ5 protein at multiple loci [182]. While the function of RecQ5 in RNAPII elongation complexes is not yet known, we favor a model in which it remains poised on the PCTD, ready to act if the polymerase encounters a situation that might induce inappropriate transcription-linked recombination (e.g., [174–179]). It will be extremely informative to analyze the involvement of transcribing RNAPII, and proteins associated with its PCTD, in repair/recombination events that contribute to genome stability.

## 6. Conclusions

After 25 years of research, much is still unknown about the CTD of RNAPII and its role in coordinating a surprising number of nuclear events with transcription. In addition to transcript elongation (RNAPII movement along the template), mRNA processing, and chromatin modification, the collection of CTD-interacting processes is now thought to also include mRNA export and DNA repair; future investigations into the links between these events and the CTD should be remarkably informative. New information on CTD phosphorylation patterns, which modulate its interactions with nuclear factors, has recently been generated through genome-wide ChIP experiments, and multiple new insights into global CTD phosphorylation have emerged. On the other hand, the complexity and nuance of the patterns of post-translational modifications are such that the actual distribution of phosphate groups along the CTD is not known for even one transcription elongation complex. Thus, while the inventory of CTD-modifying enzymes continues to expand, it is clear that we have much to learn about the ways in which they collaborate to produce modification patterns as found *in vivo*. Even so, the list of proteins known to interact with a specifically modified form of the CTD also continues to grow, expanding our knowledge of the roles played by the CTD in coordinating transcription-related processes. As our knowledge of CTD modifications and interactions expands and becomes more refined, our understanding of the “phospho-CTD cycle” and the manner in which the PCTD orchestrates the numerous events connected to the process of DNA-dependent RNA synthesis will continue to evolve.
